# Synthetic Approaches as Empirical Tools for Testing Evolutionary Theory

**DOI:** 10.1093/gbe/evag245

**Published:** 2026-10-01

**Authors:** Xueying C Li, Paco Majic, Cauã Antunes Westmann

**Affiliations:** Ministry of Education Key Laboratory for Biodiversity Science and Ecological Engineering, College of Life Sciences, Beijing Normal University, Beijing, China; Developmental Biology Unit, European Molecular Biology Laboratory, Heidelberg 69117, Germany; Institute of Bioengineering, School of Engineering, École Polytechnique Fédérale de Lausanne, Lausanne 1015, Switzerland; Swiss Institute of Bioinformatics, Quartier Sorge-Batiment Genopode, Lausanne 1015, Switzerland

**Keywords:** evolutionary theory, molecular evolution, synthetic biology, adaptive landscapes, historical contingencies, ancestral sequence reconstruction

## Abstract

Evolutionary biology has traditionally inferred process from patterns in extant organisms and the fossil record, leaving many foundational questions constrained by their historical nature. Over the past two decades, a growing family of synthetic approaches—high-throughput mapping such as deep mutational scanning, reconstructive experiments such as ancestral sequence reconstruction, controlled evolution, and synthetic biology in the narrower sense—have made it possible to test these questions directly by systematically exploring sequence space, reconstructing historical states, replaying evolution under defined conditions, and engineering biological systems. Here, we review how these approaches reshape several foundational questions: the distribution of mutational effects, the structure and navigability of fitness landscapes, the evolution of evolvability, developmental constraint, historical contingency, and the engineering of evolutionary systems. Across these domains, these experiments are exposing unexpected mechanistic detail that refines and extends classical theory—revealing, for example, how strongly mutational effects depend on environmental and genetic context, how ruggedness can coexist with broad accessibility on fitness landscapes, how genotype–phenotype maps are intrinsically biased and heterogeneous, and how random sequences carry latent functional potential that may serve as raw material for later innovation. As these technologies continue to expand the empirical reach of evolutionary biology, theory in turn sharpens the questions they are best suited to address—an iterative dialogue between experiment and theory that brings us closer to understanding how life evolves.

Significance Evolutionary biology has long faced a fundamental limitation: evolution unfolds over deep time, and we can study only the organisms and fossils that happen to have survived—leaving many central ideas about how evolution works difficult to test directly. New laboratory tools now allow scientists to build, modify, and replay evolutionary systems, providing direct experimental tests of these ideas. This review shows that such experiments are refining—and sometimes overturning—long-held views, for example, by revealing that mutational effects depend strongly on context, that many evolutionary trajectories are shaped by historical contingencies, and that random DNA frequently displays latent biological activity, implying that evolution may act not only to create function but also to suppress unwanted activity. Together, these advances shift the field from observing evolution to experimentally probing its underlying molecular mechanisms.

## Introduction

Evolutionary biology has long been characterized by a productive interplay between theory and empirical observation, with each continually informing and refining the other. Early empirical challenges to transformist ideas, such as comparisons of ancient and extant organisms, foreshadowed the more systematic synthesis achieved by Charles Darwin in *On the Origin of Species* (Darwin 1859*)*, where conceptual reasoning was tightly interwoven with diverse lines of evidence. Subsequent discoveries, notably the fossil of *Archaeopteryx*, reinforced evolutionary predictions by revealing transitional forms. Over time, major theoretical advances have provided unifying frameworks: the foundations of population genetics laid by Ronald A. Fisher (Fisher 1930), J. B. S. Haldane (Haldane 1931, 1990), and Sewall Wright (Wright 1931, 1932) formalized the roles of mutation, selection, drift, and migration, while later developments—including the neutral theory advanced by Motoo Kimura (Kimura 1962, 1968, 1983) and Tomoko Ohta (Ohta 1973), as well as models of adaptive dynamics and evolutionary game theory—extended evolutionary analysis into new domains. In parallel, empirical support has expanded across genetics, paleontology, comparative biology, biogeography, and controlled experiments, from early work in *Drosophila* to long-term microbial evolution studies. Together, these intertwined empirical and theoretical traditions have transformed evolutionary biology into a quantitatively grounded discipline capable of explaining and predicting patterns of biological diversity.

Yet the sources of empirical information have been limited by the inescapable fact that evolution is ultimately a historical process. Thus, many of the questions in evolutionary theory have been constrained by inferring what processes have underlain the observations of “design features of ‘finished products’” (Dennett 1995). The study of evolutionary processes has been greatly improved by the capacity to run evolutionary simulations using computational programs (Dennett 1995; Wagner and Altenberg 1996; Dawkins 2019; Hansen and Wagner 2023). Though useful to investigate dynamical processes and to act as proof-of-principle of potential evolutionary avenues, such simulations were still restricted to stark simplifications of biological parameters. Over the last few decades, however, new synthetic approaches—spanning high-throughput mapping, reconstructive experiments, controlled evolution, and synthetic biology in the narrower sense—have provided complementary experimental tools that allow us to revisit foundational ideas directly in the laboratory. Techniques such as high-throughput DNA sequencing, genome editing, and synthetic gene construction now enable researchers to directly test classical evolutionary hypotheses, often affirming their relevance while also challenging long-held assumptions (Crocker and Ilsley 2017; Yi and Dean 2019; Ornelas et al. 2023).

These synthetic approaches (Box 1, Fig. 1) complement inference from extant natural variation by making selected evolutionary possibilities experimentally accessible. They cannot recover extinct histories in full, but they allow researchers to reconstruct candidate historical states, systematically assay regions of genotype space, and engineer biological systems or evolutionary regimes that natural evolution has not sampled. Combined with comparative evidence, they can test whether particular genetic changes are sufficient or necessary for phenotypic differences. For example, genome editing and functional replacement have been useful to examine the genetic causes of the limblessness of snakes (Kvon et al. 2016), the eyespots of butterfly wings (Murugesan et al. 2022), the chiropatagium of bats (Schindler et al. 2025), the winglike fins of flying fish (Daane et al. 2021), or the exaggeration of sexually selected traits like the boxing first pair of legs of *Drosophila prolongata* (Audet et al. 2026).

Box 1. What are synthetic approaches to biology? Throughout this review, we use *synthetic approaches* as a broad operational term for experimental strategies that create, reconstruct, systematically perturb, or deliberately explore biological states beyond the variation directly available from observation of extant natural systems. We do not use it to imply that all such approaches belong to the disciplinary field of synthetic biology. In practice, the studies reviewed here typically lie at the intersection of several disciplines—synthetic biology (DNA synthesis, directed evolution), genetics (mutagenesis, phenotyping), genomics (sequencing technologies), and evolutionary biology (theory and modeling).

We distinguish four overlapping experimental modes, corresponding to the four categories in Fig. 1:

- **High-throughput mapping**—deep mutational scanning, combinatorial libraries, and massively parallel reporter assays that systematically explore genotype–phenotype or genotype–fitness relationships.
- **Reconstruction**—ancestral sequence reconstruction and related methods that experimentally test inferred historical states and mutational paths.
- **Controlled evolution**—experimental evolution, directed evolution, and engineered mutational regimes that manipulate evolutionary dynamics under defined conditions.
- **Synthetic biology (in the narrower sense)**—the deliberate design, construction, or redesign of biological systems or evolutionary processes, for example, engineered gene circuits, minimal or refactored genomes, altered mutation-generating machinery, expanded genetic codes, or de novo sequences.

These modes share enabling technologies—DNA synthesis, genome editing, high-throughput sequencing, and automated phenotyping—that do not by themselves define an experiment as synthetic biology: deep mutational scanning is primarily a mapping strategy, the synthesis of an ancestral or heterologous gene is an enabling step in a reconstructive experiment, and a conventional single-gene knockout remains a form of classical genetic perturbation. What unites the studies considered here is therefore not membership in a single discipline, but their use of constructed, reconstructed, or deliberately expanded biological variation to make evolutionary hypotheses experimentally tractable.

**Fig. 1.**
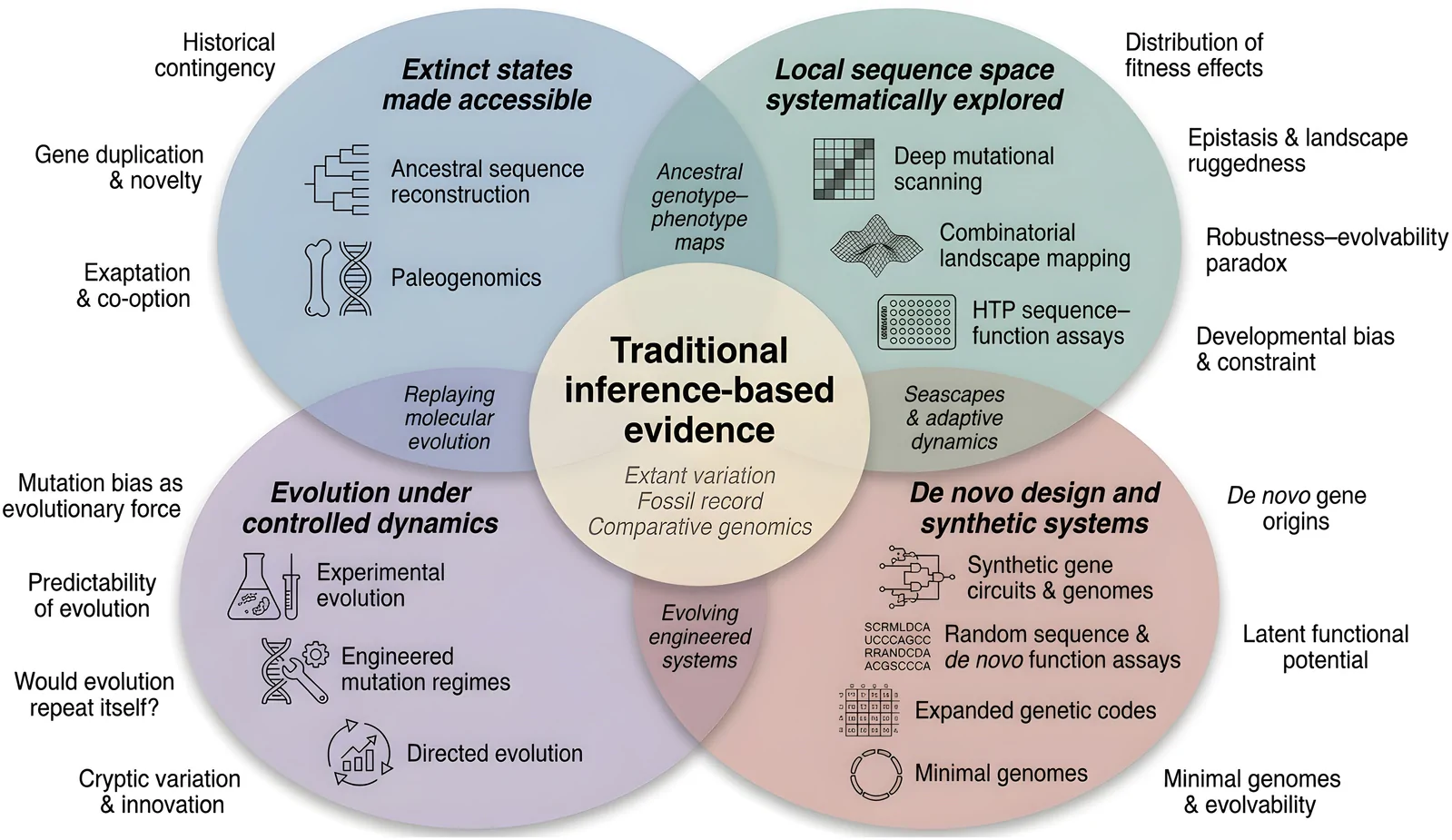
Synthetic approaches expand evolutionary access beyond natural observation. Evolutionary biology has traditionally relied on inference from extant variation, the fossil record, and comparative genomics (center). Four complementary classes of approaches now extend this empirical reach. *Extinct states made accessible* (upper left): ancestral sequence reconstruction and paleogenomics resurrect ancient molecules and genotypes, enabling direct functional tests of evolutionary history. *Local sequence space systematically explored* (upper right): deep mutational scanning, combinatorial libraries, and high-throughput sequence–function assays map genotype–phenotype and fitness landscapes around natural sequences with unprecedented resolution. *Evolution under controlled dynamics* (lower left): experimental evolution, engineered mutational regimes, and directed evolution allow populations to be replayed, redirected, or exposed to defined selective pressures, testing how mutation bias, contingency, and repeatability shape adaptive trajectories. *De novo design and synthetic systems* (lower right): synthetic gene circuits, minimal genomes, expanded genetic codes, and random-sequence assays probe functional regions of sequence space that natural evolution may never have sampled, addressing what evolution could do rather than only what it did. Overlap zones highlight questions that are most effectively addressed by combining approaches: ancestral genotype–phenotype maps emerge from coupling sequence reconstruction with systematic landscape mapping; replaying molecular evolution integrates resurrected ancestors with forward experimental evolution; seascapes and adaptive dynamics link empirical landscape characterization with controlled evolutionary experiments; de novo regulatory landscapes connect high-throughput assays with synthetic and random-sequence libraries; and evolving engineered systems combines experimental evolution with synthetic circuits and minimal genomes. Classical evolutionary questions are shown at the periphery. Together, these approaches shift the field from observing evolutionary outcomes to experimentally testing evolutionary possibilities. We use “synthetic approaches” for this broader family of strategies; synthetic biology in the narrower sense is represented most directly by the de novo design and synthetic-systems quadrant and by those controlled-evolution experiments that deliberately engineer evolutionary dynamics, whereas high-throughput mapping and ancestral reconstruction are other distinct modes within the broader category of synthetic approaches.

More broadly, these synthetic approaches are propelling evolutionary research into new empirical territory, not only by dissecting the mechanistic basis of morphological and physiological change but also by enabling direct tests of the rules governing evolutionary processes. In this way, they have become powerful tools for advancing evolutionary theory. In this review, we revisit classical questions in the field and examine how cutting-edge experimental approaches are providing fresh insights into them. Focusing on six major questions in evolutionary theory, we highlight representative examples in which these synthetic approaches bridge theoretical concepts and empirical data and emphasize how these emerging tools are reshaping our understanding of evolutionary dynamics, concluding with a forward-looking perspective on open questions and future directions.

## Foundational Questions Revisited Through Synthetic Approaches

### The Interplay Between Mutations and Selection

Genetic mutations play a central role in evolution by providing the ultimate source of heritable variation upon which natural selection and other evolutionary forces act. Despite this fundamental role, the evolutionary importance of mutations themselves—their rates, spectra, and biases—has been a recurrent point of debate. During the early twentieth century, mutationist perspectives gained prominence during the so-called eclipse of Darwinism (Huxley 1942), emphasizing the creative role of mutations in shaping evolutionary change. These views were later largely relegated by a selectionist framework, grounded in the assumptions that individual mutational effects are typically small relative to the strength of selection and that populations harbor sufficient standing genetic variation to respond rapidly to environmental change. The elucidation of the structure of DNA and the genetic code transformed our understanding of the nature of mutations and their evolutionary consequences, leading to major conceptual advances such as the neutral theory of molecular evolution, renewed interest in gene duplication as a driver of novelty, and the rise of comparative and population genomics. These molecular advances have promoted the idea of “mutation-driven evolution” (Nei 2013).

The ability to construct and assay mutations systematically under highly controlled conditions has further highlighted the evolutionary relevance of the mutational process itself. High-throughput generation of large numbers of sequence variants, their efficient introduction into biological systems, and precise manipulation of the molecular machinery that governs mutation rates and spectra now allow direct experimental interrogation of how mutational processes shape evolutionary trajectories. In combination with experimental evolution approaches, artificial genetic constructs and engineered mutational regimes make it possible to study evolutionary dynamics under defined conditions that either approximate natural systems or deliberately depart from them—for example, through altered mutation spectra, rewired regulatory circuits, or expanded genetic codes (Fig. 1).

#### The Distribution of Fitness Effects of Mutations Is Dynamic

The relative importance of selection and mutational input on the potential of affecting the evolutionary process depends primarily on (i) the rate at which mutations occur and (ii) the phenotypic and fitness effects of individual mutations. Our capacity to estimate both of these parameters has substantially improved over the last decades. In the case of the effects of mutations (for an overview of advances in mutation rate estimations, see Padilla-Iglesias and Majic 2026 [Padilla-Iglesias and Majic 2026]), high-throughput variant construction has been fundamental. Early efforts to estimate the distribution of fitness effects (DFE) of mutations relied on indirect approaches, most prominently mutation–accumulation experiments in *Drosophila* (Mukai 1964; Ohnishi 1977), bacteria (Kibota and Lynch 1996), and yeast (Lynch et al. 1993). By relaxing natural selection and allowing mutations to accumulate, these studies quantified the resulting declines in fitness and inferred that most new mutations are deleterious, with a wide distribution of effects. Parallel insights came from comparative genomics, where patterns of synonymous and nonsynonymous substitutions were interpreted through the lens of the neutral and nearly neutral theories (Kimura 1968, 1977; Ohta 1973; Eyre-Walker and Keightley 1999). Together, these classical approaches established the prevailing view that the DFE is skewed toward neutrality and deleteriousness, with beneficial mutations being rare—even though the precise shape of the distribution remained difficult to resolve.

High-throughput molecular tools have added much-needed nuance to classical interpretations of mutational effects. In particular, deep mutational scanning of designed variant libraries—a high-throughput measurement approach rather than synthetic biology in itself—enables direct, large-scale measurement of the fitness consequences of hundreds of thousands of gene variants in parallel. These studies challenge the simplistic view that most mutations are strongly deleterious, instead revealing different properties of DFEs such as (i) most single mutations are neutral or slightly deleterious, with only a small fraction being beneficial (Firnberg et al. 2014; Sarkisyan et al. 2016); (ii) the shape of the DFE depends strongly on environmental context (Hietpas et al. 2011; Hietpas et al. 2013); (iii) saturating proteins with mutations has revealed pervasive epistasis, whereby the effect of a mutation depends on the presence of others (Bank 2022; Di Bari et al. 2024); (iv) many DFEs are well described by truncated exponential distributions, such that small shifts in the mean markedly affect the probability of rare beneficial variants, highlighting the importance of the distribution's “tail” for adaptation (Eyre-Walker and Keightley 1999; Kassen and Bataillon 2006); and (v) the mean effect of new mutations often declines with increasing background fitness, suggesting that physiological and environmental constraints modulate the DFE (Ardell et al. 2024; Couce et al. 2024; Aguilar-Rodríguez et al. 2025).

Together, these results illustrate that DFEs are not static properties of mutations alone, but dynamic features shaped by molecular context, environment, and evolutionary history.

#### The Fitness Effect of Synonymous Mutations

The classical neutral theory proposed by Motoo Kimura treated most mutations at the molecular level as selectively neutral and regarded synonymous substitutions in particular as a paradigmatic example of neutral variation, given that they do not alter amino acid sequences (Kimura 1968, 1983). Synonymous changes, however, are now recognized as potentially having substantial functional and fitness consequences (Firnberg et al. 2014; Zwart et al. 2018; Lebeuf-Taylor et al. 2019; Bailey et al. 2021; Liu et al. 2021; Bao et al. 2022; Zhang and Qian 2025), by altering transcription, mRNA splicing, stability, and secondary structure, as well as translation efficiency and co-translational protein folding (Zhang and Qian 2025). However, recent evidence based on high-throughput experiments has been controversial: in yeast, many synonymous variants were found strongly non-neutral (Shen et al. 2022, 2024), although technical concerns have been raised with the finding (Kruglyak et al. 2023). Similar results have been observed in bacteria, where synonymous edits in *E. coli* can impair growth in a condition-dependent manner (Lebeuf-Taylor et al. 2019). Beyond deleterious outcomes, synonymous mutations have also been implicated in adaptation, for example, in experimentally evolved *Pseudomonas fluorescens* populations (Bailey et al. 2021). However, high-throughput genome-editing experiments in human cells supported the traditional view that synonymous mutations rarely have fitness effects (Rao et al. 2025). Therefore, a conclusive understanding about the neutrality of synonymous mutations awaits more empirical data.

#### Mutational Biases May Shape Evolutionary Trajectories

Classical population genetics often assumed mutation to be isotropic, with equal probability of change in all directions of sequence space, but this view has been overturned by accumulating evidence that mutation processes are inherently biased (eg transition–transversion asymmetries and pervasive GC→AT biases). While it was traditionally argued that natural selection would dominate over such biases except under neutrality, recent theoretical and experimental work shows that mutation bias can influence the trajectory of adaptation by shaping the pool of available variants. Some of the strongest evidence comes from experiments that deliberately engineer the mutational process itself. A clear experimental demonstration comes from work by Deepa Agashe and colleagues in *Escherichia coli*, who engineered strains with altered mutation spectra by modifying DNA replication machinery (Sane et al. 2025). By effectively rotating the direction of mutational bias, they showed that the supply and distribution of beneficial mutations depend strongly on mutation spectrum. Strains biased toward mutation types rare in the wild type accessed a broader set of beneficial mutations, whereas those exaggerating the native bias rapidly depleted accessible beneficial variation, exhibiting up to an order-of-magnitude reduction in beneficial mutations in the DFE (Sane et al. 2025). These results provide direct empirical support for the prediction that shifts in mutation bias can redirect adaptive evolution by altering which mutational paths are available, allowing populations with novel biases to access adaptive solutions that might otherwise remain unexplored.

#### Gene Duplication as a Source of Innovation

Gene duplication has long been considered a central engine of evolutionary innovation. Susumu Ohno proposed that duplication frees one gene copy from selective constraint, allowing it to accumulate mutations that may eventually confer a new function (“neofunctionalization”), while the other copy preserves the ancestral role (Ohno et al. 1968). Synthetic approaches have begun to test this scenario directly. The Schaerli group (Mihajlovic et al. 2025) constructed *E. coli* strains carrying either one or two copies of a fluorescent protein gene and evolved them under selection for altered color output. Two-copy populations indeed accumulated more mutations and tolerated greater genetic diversity, consistent with relaxed constraint, yet they did not consistently evolve the target trait faster than single-copy strains. Often, one duplicate decayed into a pseudogene before it could be repurposed, illustrating that duplication enables but does not guarantee innovation. These findings nuance Ohno's model by showing that the fate of duplicates depends on additional factors—selection regime, regulatory context, and the chance order of mutations.

A complementary framework, the escape from adaptive conflict (EAC) model, proposes that an ancestral gene performing multiple functions can be constrained by trade-offs and that duplication allows each copy to specialize and resolve this conflict (Des Marais and Rausher 2008; Sikosek et al. 2012). Support for EAC comes largely from ancestral reconstruction studies (see Section 2.5), which show how combining forward-evolution and historical approaches refines classical views of innovation. Classical models further assume that loss-of-function mutations in one copy are neutral. However, a recent study has shown that when duplicated genes interact, a defective copy can impair its partner, meaning purifying selection can maintain both copies longer than expected. For example, a deep mutational scan of a tetrameric protein found that ∼6% of harmful mutations disrupt the second copy, helping preserve redundancy over evolutionary time (Cisneros et al. 2026).

### The Navigability of Fitness Landscapes

Theoretical efforts to understand adaptation have long relied on spatial metaphors that map evolutionary change onto abstract representations of fitness. Sewall Wright's fitness landscape provided an intuitive framework in which populations move across adaptive peaks and valleys under the combined influences of mutation, selection, drift, and recombination (Wright 1931, 1932). Complementing this view, Fisher's geometric model of adaptation formalized how mutations of varying effect sizes influence fitness in high-dimensional phenotypic space, predicting that small-effect mutations are more likely to be beneficial as populations approach an adaptive optimum (Fisher 1930). Subsequent theoretical work explored how the topology of fitness landscapes influences evolutionary dynamics. Models of rugged fitness landscapes, including those developed by Kauffman, predicted that increasing epistasis and landscape ruggedness can constrain adaptive walks, trap populations on local optima, and generate complex evolutionary trajectories (Kauffman and Levin 1987; Kauffman and Weinberger 1989; Obolski et al. 2018; Krug and Oros 2024). The fitness landscape metaphor treats evolution as populations moving on a high-dimensional map, where the location corresponds to genotype and the elevation corresponds to fitness (Fisher 1930; Wright 1931, 1932). A central question is ruggedness—the prevalence of local peaks (genotypes whose single-mutational neighbors are all lower in fitness) and valleys (steps that reduce fitness)—and whether ruggedness traps populations on suboptimal solutions (Kauffman and Levin 1987; Kauffman and Weinberger 1989; Obolski et al. 2018; Krug and Oros 2024). Classic theory debated how populations could traverse valleys.

Advances in combinatorial library construction, genome editing, and high-throughput phenotyping have enabled direct empirical mapping of fitness landscape topology at unprecedented scale. For example, an exhaustive study of the antibiotic resistance enzyme dihydrofolate reductase in *Escherichia coli* quantified the fitness of over 260,000 genotypes using genome editing and competitive assays, revealing a highly rugged landscape with numerous local peaks (Papkou et al. 2023) (Fig. 2a). Despite this extensive epistasis, the global fitness optimum remained broadly accessible, with many mutational trajectories proceeding uphill without traversing deep fitness valleys (Papkou et al. 2023). Similar “rugged yet navigable” architectures have been observed in regulatory DNA, including transcription factor binding sites mapped via massively parallel reporter assays (Fig. 2b), where compensatory changes in core motifs, flanking regions, or spacing create ridgelike paths that maintain function (Westmann et al. 2024a, 2024b, 2026) (Fig. 2c). Other high-throughput studies across fluorescent proteins (Sarkisyan et al. 2016), enzymes (Firnberg et al. 2014; Weeks and Ostermeier 2023), regulatory elements (Aguilar-Rodríguez et al. 2017; Kuo et al. 2020), and viral proteins (Adams et al. 2019; Schulz et al. 2025) likewise report pervasive epistasis alongside neutral networks (Kimura 1977, 1983; Reidys and Stadler 2001) and multiple accessible optima (Pahujani and Krug 2025; Srivastava et al. 2026). Together, these findings suggest that while biological fitness landscapes are complex, their structure often permits evolutionary exploration through “multidimensional bypasses” (Wu et al. 2016), and connected ridges and plateaus, mitigating the constraining effects of local peaks. Rather than trapping populations, ruggedness may instead reduce predictability, as stochastic mutational paths can lead different populations to distinct adaptive peaks.

**Fig. 2.**
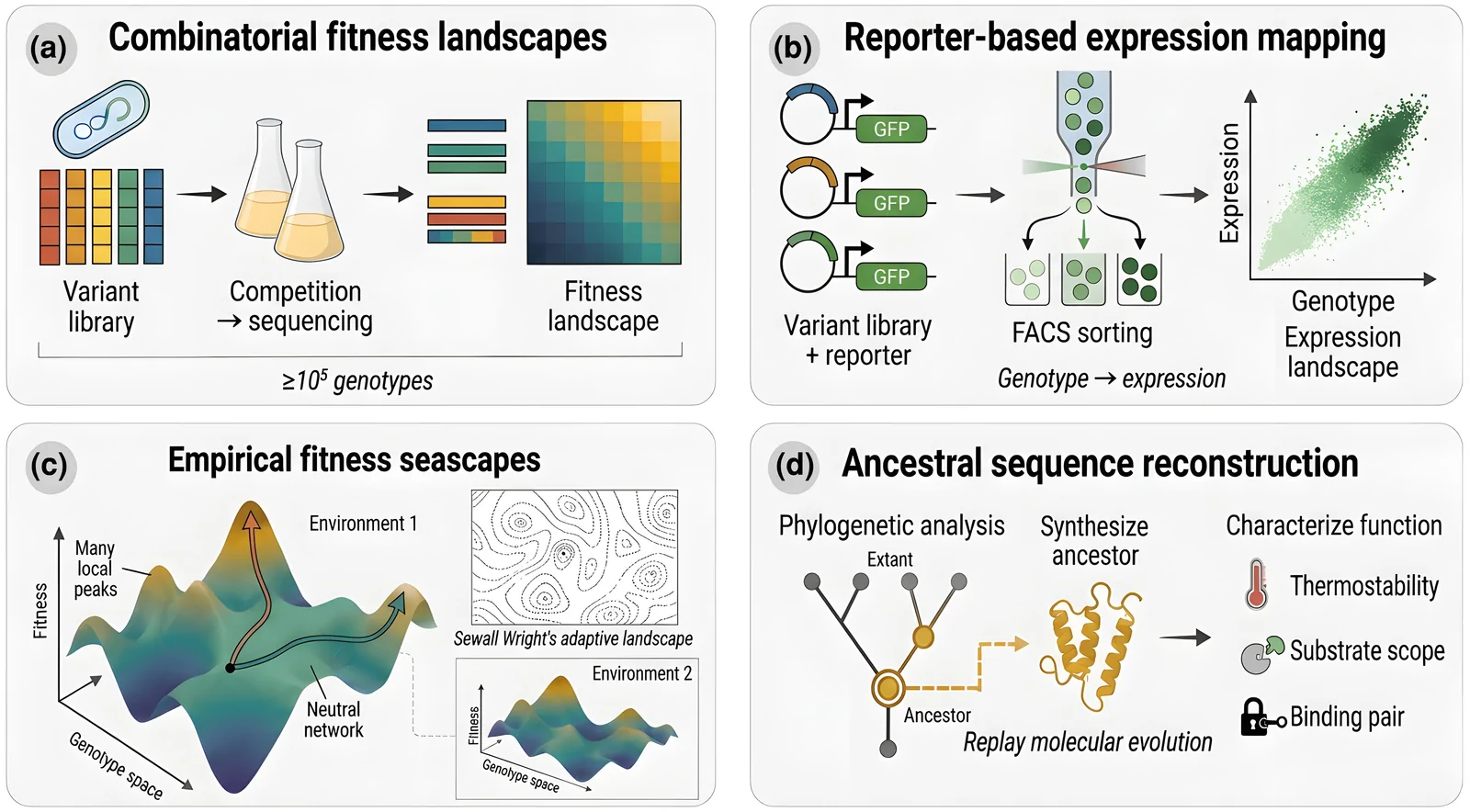
Mapping and reconstructing evolutionary possibilities. The first three panels show how high-throughput genotype–fitness maps are built and what the resulting landscapes look like, and the final panel shows how ancestral states are experimentally resurrected to replay molecular evolution. a) Combinatorial fitness landscapes. Large combinatorial variant libraries (≥10^5^ genotypes) are introduced into cells by genome editing and subjected to growth competition under defined selective conditions. Deep sequencing yields empirical fitness landscapes that quantify in vivo fitness for each genotype (Papkou et al. 2023; Aguilar-Rodríguez et al. 2025; Chattopadhyay et al. 2025). b) Reporter-based expression mapping. Libraries of regulatory sequence variants are cloned upstream of a fluorescent reporter (eg GFP), expressed in cells, and sorted by fluorescence intensity using FACS. Sequencing of each sorted bin links sequence variation to quantitative regulatory output, producing high-resolution genotype–expression landscapes (de Boer et al. 2020; Vaishnav et al. 2022; Westmann et al. 2024b, 2026). c) Empirical fitness seascapes. In contrast to Wright's original (Wright 1932) (faint gray silhouette), empirical landscapes reveal richly structured topographies with many local peaks, ridges, and neutral networks. Two trajectories from a shared starting genotype illustrate that most peaks are reachable without crossing deep valleys: a direct adaptive path (coral arrow) climbs steeply to one peak, whereas neutral exploration (teal arrow) traverses a plateau before ascending a different peak. Crucially, landscape topography is environment-dependent: the same genotype space exhibits a different fitness surface under alternative conditions (inset, Environment 2), producing fitness “seascapes” in which peaks, valleys, and accessible paths shift with external context (Mustonen and Lässig 2009). d) Ancestral sequence reconstruction. Phylogenetic analysis of extant sequences is used to infer ancestral states, which are then synthesized in the laboratory and experimentally characterized for properties such as thermostability, substrate scope, and binding partner—effectively replaying molecular evolution (Thornton 2004; Herrera-Álvarez et al. 2025).

High-throughput fitness assays in microbes have also enabled systematic characterization of genotype-by-environment (G × E) interactions, revealing dynamic “fitness seascapes” (Mustonen and Lässig 2009; Ogbunugafor et al. 2016; Li and Zhang 2018; Trubenová et al. 2019; Aubé and Landry 2024) (Fig. 2c) in which peaks and ridges shift with external conditions such as drug concentration or temperature. This is exemplified by high-throughput mutational studies of the molecular chaperone Hsp90 across diverse stressors (eg heat, salinity, oxidative stress) (Hietpas et al. 2011; Bank et al. 2016; Flynn et al. 2020), as well as by analyses of promoter and transcription factor binding site variation (Chattopadhyay et al. 2025). These studies show that both ruggedness and accessibility are environment-dependent: a given genotypic neighborhood may be highly constrained in one condition yet readily navigable in another. Accordingly, predictive power improves when models are formulated in joint G × E space, incorporating environment-dependent global epistasis. The environmental dependence of the DFE is thus mirrored at the landscape level, with context reshaping not only mutational effects but also the topography traversed during adaptation.

These inferences nevertheless require caution. High-throughput maps often measure a molecular proxy or growth phenotype in a simplified or heterologous system—for example, steroid–receptor variants assayed in yeast—and therefore cannot reproduce all of the native organism's regulatory, physiological, developmental, ecological, and population-genetic constraints. A genotype that is functional in such an assay may consequently be evolutionarily inaccessible or selectively disfavored in nature; conversely, the absence of a functional laboratory genotype from extant lineages may reflect historical contingency, incomplete exploration, or limited sampling rather than an intrinsic lack of function. Experimental estimates of genotype accessibility and of the DFE should therefore be interpreted as conditional on the genotype, host, environment, and phenotype assayed, rather than as universal properties of a sequence.

### The Evolution of Evolvability

Dawkins asked whether evolvability can itself evolve (Dawkins 2019). Essentially, organismal features can be selected such that they precondition the range of adaptive solutions that become available. Dawkins referred to this as the “evolution of evolvability”—certain features act as “evolutionary watersheds” that can occasionally “open the floodgates to future evolution.”

#### Cryptic Variation

The concept of cryptic genetic variation as an evolutionary resource gained molecular footing when Rutherford and Lindquist showed that the chaperone Hsp90 acts as a phenotypic capacitor in *Drosophila*: under stress, previously buffered genetic variation is released, producing diverse morphological phenotypes (Rutherford and Lindquist 1998). Similar capacitor behavior has since been documented for chromatin regulators, prions, and DNA methylation (Gandara et al. 2022). But can cryptic variation actually accelerate adaptation? Recent experiments have directly tested the role of cryptic variation in evolution. In one study, researchers accumulated mutations in a gene encoding a fluorescent protein in *E. coli* under conditions where those mutations had no effect on the protein's function (thus, building up cryptic variation in the population). They then challenged these lineages to evolve a new function—shifting the protein's fluorescence from yellow to green under strong selection (Zheng et al. 2019). The results were striking: populations that harbored cryptic genetic variation evolved novel adaptive phenotypes faster and reached higher fitness peaks than populations without that cryptic pre-variation (Zheng et al. 2019). The cryptically mutagenized populations explored more diverse genotypic solutions and ended up with distinct mutations, whereas the non-cryptic (unmutated) populations tended to converge on the same limited set of changes. In essence, cryptic variation “opened up” access to diverse adaptive peaks, allowing different replicates to discover otherwise inaccessible high-fitness genotypes. This empirically demonstrates a long-suspected principle: genetic variation that is initially neutral can set the stage for later evolutionary leaps when the environment shifts. It also showed that cryptic variation increases the contingency and unpredictability of evolution—different populations, even in identical conditions, took divergent paths and outcomes because they had different hidden mutations available to exploit (Zheng et al. 2019). This finding has deep theoretical implications. It affirms that the past evolutionary history (even neutral drift) can profoundly influence future adaptation (a form of evolutionary “memory”). It also provides a mechanistic basis for evolvability: robustness (tolerance of mutations) and adaptability are two sides of the same coin, where robustness allows accumulation of cryptic changes that later give adaptability when conditions change.

#### Evolvability as an Object of Selection

Evolvability itself can also be a direct target of selection. In a recent long-term experiment with the bacterium *Pseudomonas fluorescens*, Barnett et al. (Barnett et al. 2025) (2025) forced populations to repeatedly switch back and forth between two ecological states, to select for populations with greater capacity to generate changes. Over time, one lineage evolved a special kind of “hypermutable” DNA sequence in a regulatory gene: a short repeat that made it very easy for mutations to occur. This repeat acted like a genetic switch, producing rapid and reversible changes that allowed the bacteria to adapt again and again. This experiment showed, for the first time in real time, that natural selection can favor genetic features that make a lineage more adaptable, proving that evolvability is not just a by-product of evolution but a trait that can itself evolve. In a related vein, experiments on protein evolvability have revealed how selection can shape future potential. One study^74^ evolved yellow fluorescent protein under strong selection and then shifted to select a new color. The variants that emerged under strong selection were also more robust to mutations (better foldability), which allowed them to evolve the new function more quickly. Similarly, Bloom et al. (Bloom et al. 2007) showed that proteins evolving in large populations tend to become more robust and stable, improving evolvability. These findings support the idea that selective regimes—including alternation of functional pressures or variation in selection strength—can shape not only present adaptation but also the potential for future adaptation.

### Developmental Constraints

The question of evolvability is further related to the topology of genotype–phenotype maps. For much of the twentieth century, evolutionary theory treated variation as essentially random with respect to need—the modern synthesis emphasized natural selection as the creative force that shapes organisms, given raw material from mutations. However, not all phenotypic variation is equally likely: the way organisms are built (their developmental biology) can bias the variants that appear (Maynard Smith et al. 1985; Alberch 1991). Developmental processes can favor some forms over others, not only limiting what is possible, but also facilitating the capacity to adapt and diversify (Uller et al. 2018).

Genome engineering and high-throughput regulatory assays have been useful to explore this question empirically at the molecular level. A long-standing argument holds that regulatory changes drive morphological evolution because they are more modular and less pleiotropic than coding mutations (Carroll 2000; Prud’homme et al. 2006, 2007), but recent evidence suggests that cis-regulatory mutations are often more pleiotropic than previously appreciated. Deep mutational scanning is beginning to resolve this debate: DMS of the yeast *TDH3* promoter shows that cis-regulatory mutations alter expression level and noise but cause milder fitness effects and lower pleiotropy than trans-regulatory mutations (Van de Zande et al. 2023; Wittkopp 2023). By contrast, DMS in developmental contexts—made tractable by high-throughput imaging—reveals that single point mutations in *Drosophila* enhancers can have large effects on the level and pattern of target gene expression (Fuqua et al. 2020; Le Poul et al. 2020), challenging the assumption of low pleiotropy and highlighting the dense regulatory information packed into developmental enhancers.

To explore developmental constraint experimentally, deep mutational scans in developmental contexts become a useful tool. In terms of regulatory mutations—which are prime drivers of developmental evolution—it is possible to explore the effects of mutations using reporter constructs that become active in developmental contexts. Some examples using these systems have shown how developmental constraint manifests itself in highly pleiotropic enhancer mutations and the difficulty in generating new patterns with a few mutations (Fuqua et al. 2020; Le Poul et al. 2020; Galupa et al. 2023). Random sequences may have high regulatory potential in *Drosophila* embryos, but their regulatory activity may be highly contingent on preestablished chromatin accessibility (Galupa et al. 2023). More empirical evidence is still needed for evaluating the ruggedness of the fitness landscape of developmental networks, but current evidence hints that it could be highly constrained. It would also be of interest to compare developmental networks to gene-regulatory networks underlying other traits, eg metabolism or behavior, in order to understand whether high constraint is a unique property of development or a general feature of complex networks.

### The Historical Contingency of Evolutionary Change

Despite its enduring influence, the role of selection as the primary driving force of trajectories across fitness landscapes has long been debated. Gould and Lewontin famously questioned the adaptationist tendency to interpret biological traits as optimal solutions to selective problems, emphasizing instead the roles of historical contingency, developmental constraint, and correlated trait evolution (Gould and Lewontin 1979)—all of which can affect the ruggedness, dimensionality, and accessibility of fitness landscapes and can, therefore, profoundly shape evolutionary outcomes, independent of selection strength alone. In the context of this criticism, Gould famously framed this issue through the metaphor of “replaying the tape of life,” asking whether evolution would retrace similar paths if history were reset or whether chance events would lead to fundamentally different outcomes (Gould 1989). This question lies at the heart of debates about evolutionary determinism and about the extent to which selection, given similar starting conditions, leads to convergent solutions versus historically contingent trajectories (Jacob 1977).

A common challenge in evolutionary biology is that we can observe only the present, not the past—yet evolution is inherently a historical process. An interesting approach to address these questions has come from our capacity to recreate the paths evolution took, but to also synthesize the paths it could have taken. Integrating this with experimental evolution and developmental and structural approaches offers a path toward understanding how contingency, constraint, and bias jointly determine the replayability—and predictability—of evolutionary change.

Ancestral sequence reconstruction (ASR) is a powerful molecular approach that seeks to infer ancient biomolecules (DNA, RNA, proteins) from extant sequences, synthesize them in the lab, and experimentally characterize their functions (Thornton 2004; Hochberg and Thornton 2017) (Fig. 2d). In essence, ASR lets us “replay” evolution in retrospect: we can resurrect long-extinct proteins or gene sequences and examine how their properties differ from modern ones, thereby testing hypotheses about what changed and why. Among other interesting conclusions, findings from ASR have particularly informed about the role of contingency in molecular evolution.

Reconstructing a molecule's evolutionary path allows direct observation of evolutionary constraint. By recreating ancient enzymes, scientists can introduce historical mutations one by one to see how function changes, revealing epistatic interactions that constrained certain evolutionary paths and channeled others. A famous example is the evolution of cortisol specificity in glucocorticoid receptors: ASR revealed that a particular mutation required to confer high specificity was only tolerated after another “permissive” mutation occurred, explaining why evolution took the path it did (historical contingency at the molecular level) (Thornton et al. 2003; Bridgham et al. 2006; Eick and Thornton 2011; Eick et al. 2012). These kinds of studies marry well with theoretical models of sequence space and adaptive walks, providing concrete data on how many mutations, and in what order, are needed for new functions (Fig. 2d).

Ancestral reconstruction has also illuminated how gene duplication generates new function through the resolution of adaptive conflict (see Section 2.1). Reconstruction of an ancestral steroid hormone receptor before a duplication event showed that it bound multiple hormones with intermediate specificity, consistent with a single gene juggling multiple roles (Bridgham et al. 2006). After duplication, one copy acquired degenerative mutations that abolished hormone-activated signaling but preserved DNA binding, converting it into a competitive repressor that fine-tunes the activity of its sister copy (Bridgham et al. 2006). This division of labor resolved an apparent regulatory conflict and demonstrated that ostensibly deleterious loss-of-function mutations can drive innovation when they occur in a duplicated background (Bridgham et al. 2006). Such studies provide direct experimental support for the EAC model and show how experimental resurrection of ancestors complements forward-evolution experiments in dissecting the mechanistic origins of new functions.

Ancestral reconstruction has also allowed the mapping of ancient genotype–phenotype relationships. Herrera-Álvarez et al. (Herrera-Álvarez et al. 2025) generated comprehensive libraries of ancestral transcription factors and their DNA response elements, assaying all possible combinations at historically variable sites. They found that the resulting genotype–phenotype maps were strongly biased: some phenotypes were far more likely to appear than others (anisotropy), and the set of phenotypes accessible from one genotype differed greatly from those around another (heterogeneity). These intrinsic biases steered ancient proteins toward the lineage-specific DNA-binding specificities that later evolved, showing that the structure of genotype–phenotype maps themselves can act as causal determinants of evolutionary outcomes. This work extends the fitness landscape framework by demonstrating that ruggedness and accessibility are not only properties of extant molecules, but also of the ancestral maps that shaped long-term evolutionary history.

Beyond informing molecular evolution, ASR has also been used to infer ancient environmental conditions. One study interpreted the thermostabilities of reconstructed enzymes from cyanobacteria and land plants as broadly consistent with long-term cooling of Earth's photic zone, from approximately 75 °C three billion years ago to approximately 35 °C by the Devonian, complementing geological paleotemperature estimates (Garcia et al. 2017). However, the use of ancestral proteins as paleothermometers remains debated (Trudeau et al. 2016; Wheeler et al. 2016): reconstructed stability can be inflated by ancestral-state uncertainty and consensus-like reconstruction bias, and high stability may reflect selective pressures other than ambient temperature—even comparatively recent ancestral proteins can be highly stable, so thermostability provides at most indirect evidence that must be weighed against geological proxies rather than treated as a direct estimate of ancient temperature. Overall, ASR gives evolutionary biologists a remarkable time machine: instead of merely inferring the past, we can experiment on it. The insights from these resurrected molecules often uphold classical narratives (eg confirming a predicted ancestral function) but also add layers of nuance (eg revealing previously unknown intermediate states or latent functions). By directly observing “missing links” at the molecular level, we enrich evolutionary theory with a more detailed understanding of how new biological functions and complexities have arisen through deep time.

## Engineering Evolutionary Systems: The Distinctive Contribution of Synthetic Biology

Beyond high-throughput measurement of natural sequence variation, synthetic biology offers a distinct empirical mode: deliberately designing, constructing, or redesigning biological systems and evolutionary processes and observing how evolution acts on them. Three classes of approach have proven especially productive.

Synthetic gene circuits and minimal genomes provide controlled platforms for evolutionary experimentation (Schaerli et al. 2018; Santos-Moreno et al. 2023). For example, the study of synthetic genetic circuits in *E. coli* has shown that certain gene-regulatory network topologies are more evolvable toward specific functions than others (Schaerli et al. 2018; Santos-Moreno et al. 2023) (Fig. 3a). By introducing engineered regulatory circuits or metabolic pathways into living cells, researchers can ask whether selection tunes their components toward predicted optima, thereby probing principles of adaptation in tractable systems (Renda et al. 2014; Nørholm 2019; Castle et al. 2021). The assembly of a minimal bacterial genome of approximately 473 genes (JCVI-syn3.0) provides a stripped-down chassis in which to ask whether selection regenerates discarded complexity (Gibson 2014; Hutchison et al. 2016; Moger-Reischer et al. 2023; Sandberg et al. 2023). Adaptive evolution of cells with such a reduced genome produced substantial fitness gains within several hundred to a few thousand generations (Moger-Reischer et al. 2023; Sandberg et al. 2023) (Fig. 3b). On the eukaryotic side, the synthetic yeast genome project *Saccharomyces cerevisiae* 2.0 (Richardson et al. 2017; Dai et al. 2020; James et al. 2025) incorporates scrambleable genome segments designed to allow controlled rearrangement under selection. Such systems test classical questions about the evolution of robustness, modularity, and genome architecture in regimes that natural genomes do not readily afford.

**Fig. 3.**
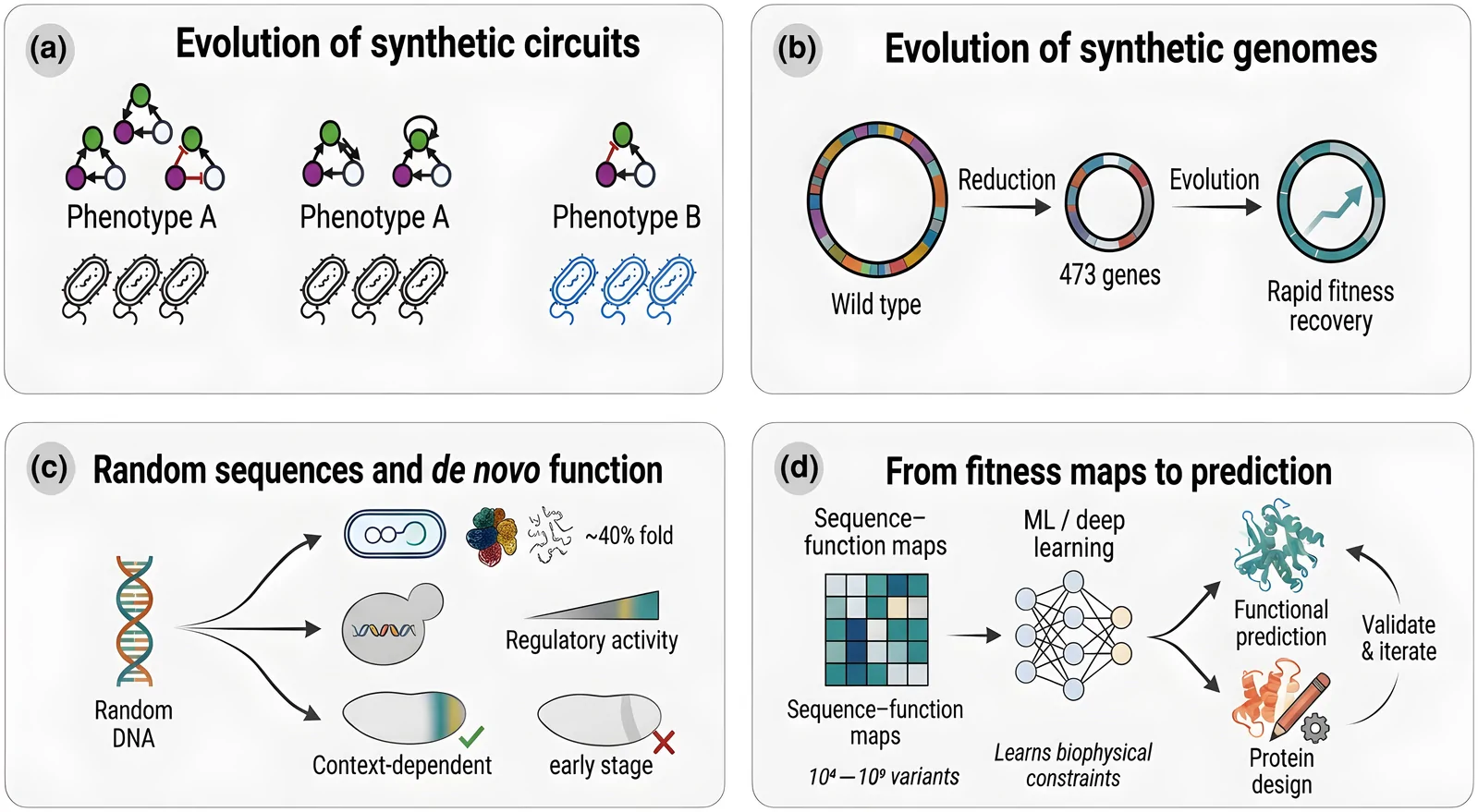
Engineering and predicting evolutionary systems. Synthetic systems reveal which evolutionary outcomes are accessible (a), how radically reduced genomes recover fitness through compensatory evolution (b), and what latent functional potential random sequences hold (c), while high-throughput maps feed models that forecast evolution (d). a) Evolution of synthetic circuits. Synthetic gene-regulatory networks with distinct topologies are introduced into cells and evolved under selection (Schaerli et al. 2018). Cell color denotes phenotype and the internal circuit diagram denotes regulatory architecture: the left and middle cells share the same color (Phenotype A) but contain different circuits, illustrating that distinct architectures can converge on the same phenotype, whereas the right cell has a different color (Phenotype B) produced by alternative wiring. Together these demonstrate that network topology constrains which evolutionary outcomes are accessible. b) Evolution of synthetic genomes. A wild-type bacterial genome is radically reduced to a minimal genome of approximately 473 genes (Hutchison et al. 2016) and then subjected to experimental evolution (Moger-Reischer et al. 2023). Rapid fitness recovery demonstrates that even highly simplified genomes retain substantial capacity for adaptation. c) Random sequences and de novo function. Random DNA sequences, never exposed to natural selection, are tested for biological activity across three systems. In *E. coli*, ∼40% of random polypeptides adopt compact, folded conformations (Tretyachenko et al. 2022). In yeast, random sequences frequently contain transcription factor binding sites that drive measurable regulatory activity (de Boer et al. 2020). In *Drosophila* embryos, random sequences can drive reporter expression at late developmental stages but not at early stages, revealing that functional potential is widespread but context-dependent (Galupa et al. 2023). d) From fitness maps to prediction. High-throughput sequence–function maps encompassing 10^4^ to 10^9^ variants feed machine-learning and deep-learning models that learn latent biophysical constraints. These models enable functional prediction and protein design, with iterative experimental validation closing the loop between prediction and observation (Vaishnav et al. 2022; Wagner 2024; Sandhu et al. 2025).

Engineered mutators decouple variation from natural mutation processes (Molina et al. 2022). Programmable in vivo platforms such as EvolvR (Halperin et al. 2018; Molina et al. 2022) can target hypermutation to specific loci using CRISPR-guided DNA polymerases, while systems such as TF-HighEvo (Li et al. 2024) in *Drosophila* fuse a transcription factor to an activation-induced deaminase to concentrate germline mutations around its binding sites, raising local mutation rates roughly tenfold and generating heritable phenotypic diversity. These tools allow direct experimental control over the supply, location, and spectrum of variation, providing tests of how mutation rate and bias shape adaptation in regimes that natural mutagenesis cannot easily produce.

Random sequences interrogate the null expectation for biological function. What fraction of sequence space is functional, and how readily can selection convert noise into signal (Fig. 3c)? Random RNAs and peptides expressed in *E. coli* often show measurable bioactivity, modulating growth in unexpected ways (Neme et al. 2017). Random sequences also display surprisingly high structural propensity: ∼40% of random 105-residue proteins adopt compact, protease-resistant conformations whether built from the full 20-amino-acid alphabet or from a reduced 10-residue prebiotic set (Tretyachenko et al. 2022), and de novo designs using only the ten “early” amino acids can reconstruct ancient protein folds with thermostability and mutational robustness comparable to their full-alphabet counterparts (Giacobelli et al. 2025); the canonical alphabet itself appears to have been shaped by these foldability constraints, since prebiotically abundant noncanonical residues yield polypeptides that fold less reliably than the early canonical set (Makarov et al. 2023).

Random DNA sequences have also been used to study the potential of de novo evolution of gene-regulatory elements. In bacteria, roughly 10% of random short DNA sequences placed upstream of a reporter in *E. coli* function as promoters without any evolution, and 60% become active after a single mutation (Yona et al. 2018). Biophysical models calibrated on random mutant libraries reach similar conclusions, predicting that ∼20% of arbitrary sequences drive measurable expression and that ∼82% of inactive sequences lie one mutation away from a functional σ^70^ promoter (Lagator et al. 2022). This propensity for de novo regulatory emergence is even greater in random DNA than in extant genomic sequence: a recent massively parallel reporter assay found that promoters arise roughly threefold more readily from random DNA than from native *E. coli* sequences, because the genome contains fewer proto-binding sites for transcriptional activators than random DNA does (Fuqua and Wagner 2025). Screens of approximately 100 million random promoters in yeast similarly show that random sequences frequently contain transcription factor binding motifs and span much of the natural expression range (de Boer et al. 2020; Vaishnav et al. 2022). In *Drosophila* embryos, random sequences readily drive reporter expression at mid- and late-stage development but rarely in early embryos, where preestablished chromatin accessibility constrains regulatory activity (Galupa et al. 2023). Together, these results suggest that, in many contexts, evolution must act as much to suppress unwanted activity as to create new function—and that natural genomes may even bear the signature of selection trimming away regulatory potential that random DNA possesses by default—reframing a long-standing question about the origins of regulatory novelty.

## Conclusion

Bringing together these threads, it is evident that we are in a golden age of empirical evolutionary biology that is deeply informed by and informing theory. The dialogue between past and future—between classical ideas and modern approaches—is enriching both. We find that many foundational concepts were essentially on the right track, but reality is often more quantitatively nuanced and interconnected than early models assumed. What do these advances provide for the future of evolutionary theory? The progression from measuring sequence–function relationships (Fig. 2a and b) to characterizing fitness seascapes (Fig. 2c) and reconstructing evolutionary history (Fig. 2d), to testing architectural constraints and de novo function with synthetic systems (Fig. 3a to c), to predicting evolutionary outcomes (Fig. 3d) mirrors the broader intellectual trajectory of the field: from cataloguing evolutionary possibilities to forecasting which of those possibilities will be realized. These approaches should not be collapsed into a single discipline: high-throughput mapping, reconstructive experiments, and synthetic biology provide distinct but increasingly convergent ways of making evolutionary possibilities experimentally accessible.

As empirical fitness landscapes and genotype–phenotype datasets expand, machine-learning approaches are beginning to capture latent biophysical constraints and generalize across sequence space (Vaishnav et al. 2022; Sandhu et al. 2025), and deep neural models trained on exhaustive landscape data can infer fitness for unmeasured genotypes and guide experimental design (Wagner 2024). Yet these advances also caution against overconfidence: replay experiments and large-scale maps consistently show that the order of mutations, clonal competition, and environmental fluctuations make evolution partly unpredictable (Wortel et al. 2023). A more realistic goal is probabilistic forecasting—estimating which outcomes are most likely and how probable different evolutionary paths are, rather than predicting single trajectories.

The horizon of tools keeps expanding. Long-read sequencing and ancient DNA may allow us to directly observe evolution over longer timescales (thousands of years) in natural populations, bridging the gap between lab time and geological time. CRISPR-based lineage tracking in multicellular organisms could open experimental evolution to plants and animals, not just microbes—one could envision tracking somatic evolution or virus evolution within a host in unprecedented detail (Askary et al. 2025). Barcode-based approaches are already providing analogous resolution in microbes: STAMPR, for example, uses highly diverse sequence tags to quantify infection bottlenecks and patterns of pathogen dissemination (Hullahalli et al. 2021), and related lineage-tracing strategies should increasingly resolve bacterial population histories across space and time. These will pose new theoretical questions about heterogeneous environments and multilevel selection (eg cell-level vs. organism-level fitness) and allow their evolutionary implications to be explored empirically (Majic et al. 2025). Synthetic biology also offers the possibility of constructing a minimal life form and seeing what directions evolution takes it in various worlds, akin to simulating alternative histories. Furthermore, as empirical fitness landscapes and genotype–phenotype datasets expand, the prospect of predictive evolution—anticipating likely adaptations in pathogens, tumors, or crops—moves from speculation toward practice.

## Data Availability

No data was generated for this manuscript. All references and materials are available from the corresponding publishers.
